# Endothelial Nitric Oxide Synthase Dimerization Is Regulated by Heat Shock Protein 90 Rather than by Phosphorylation

**DOI:** 10.1371/journal.pone.0105479

**Published:** 2014-08-25

**Authors:** Weiguo Chen, Hongbing Xiao, Alicia N. Rizzo, Wei Zhang, Yifeng Mai, Meng Ye

**Affiliations:** 1 Department of Medicine, The Affiliated Hospital of Medical School, Ningbo University, Ningbo, Zhejiang Province, China; 2 Division of Pulmonary, Critical Care, Sleep and Allergy, Department of Medicine, University of Illinois College of Medicine, Chicago, Illinois, United States of America; 3 Department of Pharmacology, University of Illinois at Chicago, Chicago, Illinois, United States of America; University of Illinois College of Medicine, United States of America

## Abstract

Endothelial nitric oxide synthase (eNOS) is a multifunctional enzyme with roles in diverse cellular processes including angiogenesis, tissue remodeling, and the maintenance of vascular tone. Monomeric and dimeric forms of eNOS exist in various tissues. The dimeric form of eNOS is considered the active form and the monomeric form is considered inactive. The activity of eNOS is also regulated by many other mechanisms, including amino acid phosphorylation and interactions with other proteins. However, the precise mechanisms regulating eNOS dimerization, phosphorylation, and activity remain incompletely characterized. We utilized purified eNOS and bovine aorta endothelial cells (BAECs) to investigate the mechanisms regulating eNOS degradation. Both eNOS monomer and dimer existed in purified bovine eNOS. Incubation of purified bovine eNOS with protein phosphatase 2A (PP2A) resulted in dephosphorylation at Serine 1179 (Ser1179) in both dimer and monomer and decrease in eNOS activity. However, the eNOS dimer∶monomer ratio was unchanged. Similarly, protein phosphatase 1 (PP1) induced dephosphorylation of eNOS at Threonine 497 (Thr497), without altering the eNOS dimer∶monomer ratio. Different from purified eNOS, in cultured BAECs eNOS existed predominantly as dimers. However, eNOS monomers accumulated following treatment with the proteasome inhibitor lactacystin. Additionally, treatment of BAECs with vascular endothelial growth factor (VEGF) resulted in phosphorylation of Ser1179 in eNOS dimers without altering the phosphorylation status of Thr497 in either form. Inhibition of heat shock protein 90 (Hsp90) or Hsp90 silencing destabilized eNOS dimers and was accompanied by dephosphorylation both of Ser1179 and Thr497. In conclusion, our study demonstrates that eNOS monomers, but not eNOS dimers, are degraded by ubiquitination. Additionally, the dimeric eNOS structure is the predominant condition for eNOS amino acid modification and activity regulation. Finally, destabilization of eNOS dimers not only results in eNOS degradation, but also causes changes in eNOS amino acid modifications that further affect eNOS activity.

## Introduction

Endothelial nitric oxide synthase (eNOS) is an important enzyme that exists in many tissues and organs. It serves to generate nitric oxide (NO), which regulates vascular tone, angiogenesis, and multiple inflammatory signaling pathways [1], [2], [3]. Two identical eNOS monomers can associate to form a dimer. Each eNOS monomer has a reductase domain and an oxygenase domain. The reductase domain contains nicotinamide adenine dinucleotide phosphate (NADPH), flavin adenine dinucleotide (FAD) and flavin mononucleotide (FMN) binding domains. The oxygenase domain contains tetrahydrobiopterin (BH_4_), heme, heat shock protein 90 (Hsp90) and zinc binding domains [4], [5]. Additionally, eNOS contains binding domains for the Calcium (Ca^2+^)/Calmodulin (CaM) complex, which enhances electron transfer from the reductase domain to the heme center of the oxygenase domain. Donation of NADPH electrons from the reductase domain to the oxygenase domain allows the electrons to interact with the heme and BH_4_ binding domains, which is necessary to catalyze the reaction of L-arginine to L-citrulline that generates NO [4].

The ability of eNOS to generate NO depends on many factors, including the presence of multiple co-factors [6] and the phosphorylation status of several critical amino acids including Serine 1177 (Ser1177) and Threonine 495 (Thr495) in human eNOS (corresponding to Serine 1179 (Ser1179) and Threonine 497 (Thr497) in bovine eNOS) [7]. Phosphorylation of eNOS at Ser1177 enhances eNOS activity 2–3 fold. The mutation of Ser1177 to aspartic acid, which mimics a negative charge on the 1177 position, also increases eNOS activity 2–3 fold [8]. Additionally, previous studies have demonstrated that bradykinin induces dephoshporylation of Thr497, which enhances Ca^2+^/CaM binding to eNOS and ultimately increases eNOS activity and NO generation. However, the implications of these phosphorylation events on eNOS structure and degradation have not yet been investigated [9].

It has been established that eNOS dimerization is necessary for normal eNOS function [6]. In eNOS dimers, one eNOS monomer reductase domain donates an electron to the other eNOS monomer's heme center of the oxygenase domain, where it is able to catalyze the conversion of L-arginine to L-citrulline, generating NO. In its monomeric form, eNOS is unable to deliver electrons to the heme center of another eNOS monomer and the electrons are instead transferred to a different position on the same eNOS monomer. This allows for the interaction with oxygen and the formation of superoxide (O_2_^−^) free radicals [10], [11]. Notably, changes in the eNOS dimer∶monomer ratio have been observed in diseases such as diabetes and myocardial infarction [12], [13]. These important findings have spurred recent interest in determining the mechanisms of eNOS dimerization and identifying molecules that may enhance this process.

It was recently demonstrated that BH_4_, a co-factor of eNOS, can restore eNOS dimerization and partially restore its function, suggesting that this molecule may be a novel target for translational research with implications in multiple diseases [12], [14], [15], [16]. Some studies have reported that the co-factor L-arginine also increases eNOS dimerization, but others have not observed this effect [14], [16]. Ca^2+^ and CaM have been reported to alter eNOS structure and activity without changing the eNOS dimer∶monomer ratio [17], [18]. Additionally, some eNOS-associated proteins play important roles in maintaining eNOS in the dimerized state. These include heat shock protein 90 (Hsp90), which has been reported to restore eNOS dimer structure and function [10], [19]. Also, a decrease in the membrane-localized protein caveolin-1 is associated with a decrease in eNOS dimers in the cellular membrane fraction [20]. However, the exact mechanism by which eNOS co-factors and associated proteins affect eNOS dimerization is not fully understood.

In addition to the effects of dimerization on eNOS activity, the mechanisms of eNOS degradation are also poorly understood. Recent studies have demonstrated that another NOS family member, inducible nitric oxide synthase (iNOS), is more stable in its dimer form than its monomer form, and iNOS monomers were found to be ubiquitinated and degraded by the 26S proteasome [21], [22]. The substantial structural and functional similarities between iNOS and eNOS suggest that eNOS may be degraded by a similar mechanism. However, despite the multiple key roles of eNOS, the mechanisms underlying its dimerization and degradation are not completely understood. Specifically, it has not yet been established if there is an optimal ratio of eNOS dimer∶monomer *in vitro* or *in vivo*. Additionally, the potential clinical implications of altering the eNOS dimer∶monomer ratio have not yet been explored.

In the current study, we investigated the mechanisms of eNOS degradation and regulation in bovine aorta endothelial cells (BAECs). Additionally, we investigated the effect of Ser1179 and Thr497 dephosphorylation on eNOS dimer stability. We found that similar to iNOS, eNOS is covalently linked to ubiquitin and degraded through 26S proteasome. Inhibition of the 26S proteasome results in eNOS monomer accumulation and thus a decrease in the eNOS dimer∶monomer ratio in BAECs. Neither Ser1179 nor Thr497 dephosphorylation was found to change the dimer∶monomer ratio of purified bovine eNOS; however, these dephosphorylation events were found to decrease eNOS activity. In contrast, Ser1179 phosphorylation on eNOS dimer, but not on eNOS monomer, indicates that eNOS dimer structure is necessary for this modification. Additionally, inhibition of Hsp90 results in a decrease in eNOS dimer and monomer content in BAECs, which accompanied both Ser1179 and Thr497 dephosphorylation.

## Materials and Methods

### Materials

Bovine aortic endothelial cells (BAECs) and cell culture media were obtained from Lonza. (Walkersville, MD). A monoclonal antibody against eNOS and the antibodies against eNOS Ser1179 phosphorylation and eNOS Thr497 phosphorylation were obtained from Upstate Biotechnology Inc. (Lake Placid, NY). Protein phosphatase 2A (PP2A) and Protein phosphatase 1 (PP1) kit was obtained from Millipore (Billerica, MA). N-methyl-D-glucamine dithiocarbamate/ferrous sulfate (MGD-Fe) and 5-(Diethoxyphosphoryl)-5-methyl-1-pyrroline-N-oxide (DEPMPO) were purchased from Alexis. CaM, NADPH, L-arginine, BH_4_, N-nitro-L-arginine methyl ester (L-NAME), and other reagents were purchased from Sigma Chemical Co. (St. Louis, MO), unless otherwise indicated. Both siRNA scramble control (siRNA control) and smart pool siRNA of heat shock protein 90 (siHsp90) were purchased from Dharmacon (GE Health Life Science, Pittsburgh, PA).

### Protein Purification

Bovine eNOS cDNA that encodes the full sequence was overexpressed in E. coli BL21 (DE3). The recombinant protein was purified in the presence of saturated BH_4_ as described previously [14].

### Determination of SDS-resistant eNOS dimers and monomers

SDS-resistant eNOS dimers and monomers were assayed using low-temperature SDS-PAGE (LT-PAGE) under reducing conditions as described previously, with minor modifications [23]. Briefly, after treatment, purified eNOS or cell samples were subjected to low temperature SDS-PAGE under reducing condition (2.5% 2-mercaptoethanol) to resolve dimers and monomers. The protein bands of dimers and monomers were visualized by Coomassie blue staining. The signal intensity of the protein band was digitalized and quantified using an AlphaImager high performance gel documentation and image analysis system, model 3300 (Alpha Innotech Co. San Leandro, CA) [14].

### eNOS dephosphorylation experiment

The purified eNOS was dephosphorylated by incubation with PP2A or PP1 according to the manufacturer's instructions and the following references [24], [25]. The reaction system contained 20 mM MOPS pH 7.5, 0.15 M NaCl, 60 mM β-mercaptoethanol, 1 mM MgCl_2_, 1 mM EDTA, 0.1 mM MnCl_2_, 1 mM DTT, 10% glycerol, and 0.1 mg/ml serum albumin. After incubation of purified eNOS with PP2A or PP1 at room temperature or on ice for 2 hours, the resulting mixtures were run on low-temperature gels to determine eNOS dimer changes [26].

### Measurement of eNOS superoxide (O_2_^−^) generation and NO generation using EPR

Superoxide (O_2_^−^) generation by purified eNOS was measured by EPR spectroscopy using the described methods with minor modifications [27]. Briefly, spin-trapping measurements of oxygen free radicals were performed in 50 mM Tris-HCl buffer, pH 7.6, containing 0.5 mM NADPH, 0.5 mM Ca^2+^, 10 µg/ml calmodulin, 2 µg purified eNOS, and 20 mM spin trap DEPMPO. EPR spectra were recorded in a disposable micropipette (50 µL, VWR Scientific) at room temperature (23°C) with a Bruker EMX spectrometer operating at X-band with a high sensitive (HS) cavity (Brucker Instrument, Billerica, MA) using a modulation frequency of 100 kHz, modulation amplitude of 0.5 G, microwave power of 20 mW, and microwave frequency of 9.863 GHz. The settings were as follows: central magnetic field 3510.0 G, sweep width 140.0 G, time constant 163.84 ms, sweep rate 40.96 ms, and receiver gain 2×10^6^. The NO generation by eNOS was measured by Fe^2+^-MGD assay [28]. The reaction system contained 5 µM reduced hemoglobin, 2 µg purified human eNOS, 0.2 mM DTT, 1 mM CaCl_2_, 1 µg/ml CaM, 5 µM BH_4_, 200 µM NADPH, 50 mM HEPES buffer, pH 7.4 in a total volume of 100 µl. The reaction was initiated by the addition of 100 µM L-arginine. The NO generation from eNOS was measured Fe^2+^-MGD-NO adduct by EPR with modification [29]. The EPR recording parameters were: microwave frequency 9.8 GHz, center field 3440 G, modulation amplitude 6 G, sweep width 100 G, receiver gain 1×10^5^, microwave power 10 mW, total number of scans 121, sweep time 10 s, and time constant 20 ms.

### Statistical analysis

Student's t-test was used to compare the means of data from two experimental groups and ANOVA followed by Tukey's post-hoc test was used to compare amongst multiple groups with significant differences considered at (P<0.05). Results are expressed as means ± SE.

## Results

**Figure 1:**
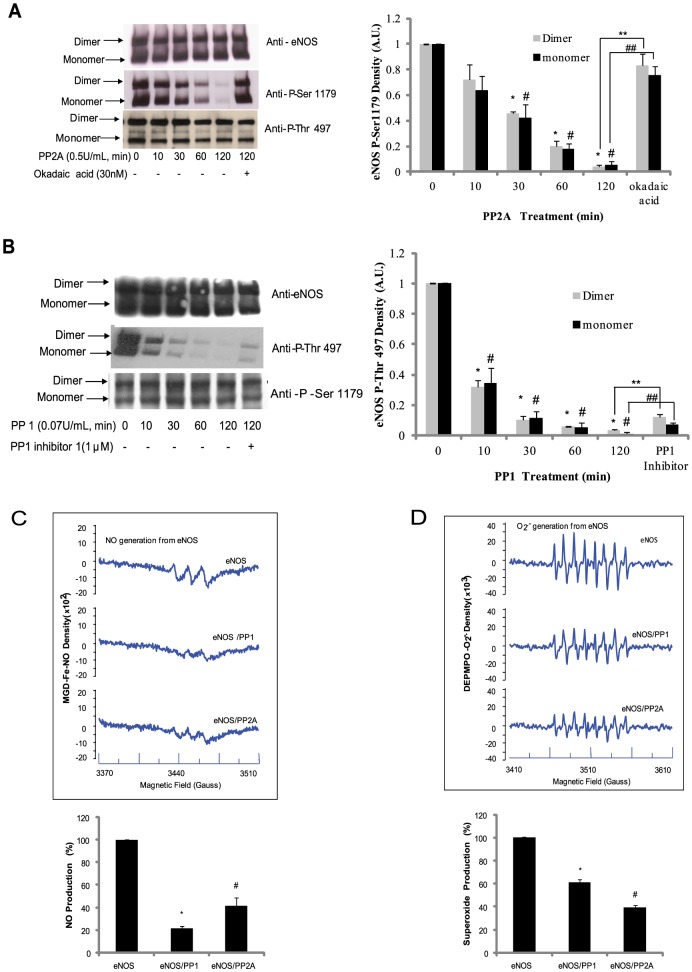
Dephosphorylation of Serine 1179 and Threonine 497 changed eNOS activity without affecting eNOS structure. (A) 2 µg purified bovine eNOS was incubated with PP2A for indicated time in the absence or presence of 30 nM okadaic acid. The eNOS sample was run on SDS-PAGE gel at low temperature. Ser1179 and Thr497 phosphorylation status was assessed. The Ser1179 phosphorylation signal in eNOS dimer and monomer was determined by densitometry and represented a bar graph. *P<0.05 by one way ANOVA relative to control for dimers and #P<0.05 for monomers. **P<0.05 for okadaic acid with PP2A treatment vs. PP2A alone for dimers and ## P<0.05 for monomers. (B) 2 µg purified bovine eNOS was incubated with PP1 in the presence or absence of 1 µM PP1 inhibitor 1. The mixtures were run on SDS-PAGE gel at low temperature and blotted with indicated antibodies *P<0.05 by one way ANOVA relative to control for dimers and #P<0.05 for monomers. **P<0.05 PP1 with PP1 inhibitor treatment vs PP1 alone for dimers and ##P<0.05 for monomers. (C) 2 µg purified eNOS was incubated PP2A or PP1 for 2 hours on ice and then Ca^2+^, CaM and L-arginine, NADPH and MGD-Fe^2+^ was added and the resulting samples were run EPR. The MGD-Fe^2+^-NO EPR signal was recorded as described in the material and methods. The graph shows typical eNOS characteristics with PP1 and PP2A inhibitory activity (* and # P<0.01 respectively compared with control by one way ANOVA. n = 4). (D) After PP2A or PP1 treatment, DEPMPO, Ca^2+^/CaM and NADPH were added to the mixture at indicated concentration and the DEPMPO-OH adduct EPR signal was recorded as described in the material and methods. The bar graph shows typical eNOS superoxide generation capacity with PP1 and PP2A inhibitory effects (* and # P<0.01 compared with control by one way ANOVA with n = 4).

Debate over the functional significance of eNOS dimers versus monomers is ongoing; however there is a growing consensus that eNOS dimers are the functionally active form [14], [23]. To address this hypothesis, we overexpressed bovine eNOS in E. coli and purified eNOS by fast protein liquid chromatography (FPLC). The purified eNOS existed as both monomer (135 kDa) and dimer (260 kDa) on low temperature gel (Figure 1A and 1B top panel). The ratio of eNOS monomer and dimer in purified eNOS from E. coli was 1∶1. The high content of eNOS monomer in purified eNOS could be due to lack of eNOS co-factors, such as Ca/CaM, and L-arginine. Additionally, E. coli may also lack eNOS degradation systems, such as the proteasome. Previous studies have demonstrated that Ser1179 phosphorylation (human eNOS Ser1177) is critical to eNOS activity [30]. Specifically, phosphorylation of Ser1179 or the mutation of Ser1179 to aspartic acid results in a 2–3 fold increase in eNOS activity [31]. However, it is still unclear whether eNOS phosphorylation alters its structural and functional properties, including eNOS dimer structure. To address this, we incubated purified bovine eNOS with PP2A to induce dephosphorylation of Ser1179 and measured the ratio of eNOS dimer∶monomer. As expected, PP2A treatment caused a time-dependent dephosphorylation Ser1179 of eNOS (Figure 1A middle panel); however, Ser1179 dephosphorylation did not alter the ratio of eNOS dimer∶monomer (Figure 1A top panel). Moreover, PP2A-treatment did not change the phosphorylation status of Thr497 (Figure 1A bottom panel). Subsequently, okadaic acid, a PP2A inhibitor, was found to prevent PP2A-mediated Ser1179 dephosphorylation without changing the eNOS dimer∶monomer ratio, indicating that the Ser1179 phosphorylation mediated enhancement in eNOS activity does not occur via changes in eNOS dimerization.

It has also been established that Thr497 may play an important role in eNOS function. Specifically, phosphorylated Thr497 can hinder Ca^2+^/CaM complex binding to eNOS, thus inhibiting eNOS activity [32]. Additionally, it was recently reported that bradykinin dephosphorylates Thr497 and initiates Ca^2+^/CaM complex binding to eNOS [9]. Therefore, we next investigated whether Thr497 dephosphorylation alters the eNOS dimer∶monomer ratio. Treatment of purified bovine eNOS with 0.07 U/mL protein phosphatase 1 (PP1) was found to cause a time-dependent dephosphorylation of Thr497 (Figure 1B middle panel). Similar to the effects of protein phosphatase 2A (PP2A) on Ser1179, we found that PP1 induced-dephosphorylation of eNOS Thr497 did not change the ratio of eNOS dimer and monomer (Figure 1B top panel). Furthermore, incubation of 1 µM protein phosphatase 1 inhibitor 1 (PP1 inhibitor 1) with PP1 and eNOS together partially conserved the phosphorylation of Thr497. Additionally, PP1 did not change Ser1179 phosphorylation density of purified eNOS (Figure 1B bottom panel).

Next we sought to determine the effects of these phosphorylation events on eNOS activity. Using MGD-Fe^2+^ as a spin-trap to bind to NO, eNOS activity was determined after incubation of eNOS with PP1 or PP2A for two hours. The EPR spectra demonstrated that both PP2A and PP1 treatment also decreased NO generation from eNOS compared to control (Figure 1C). Due to the fact that the generation of NO and superoxide have similar mechanism, we sought to investigate the effect of Ser1179 and Thr497 phosphorylation on superoxide generation. We found that both Ser1179 and Thr497 dephosphorylation by PP1 or PP2A caused a decrease in eNOS derived DEPMPO-OH signal compared to the control (Figure 1D).

**Figure 2:**
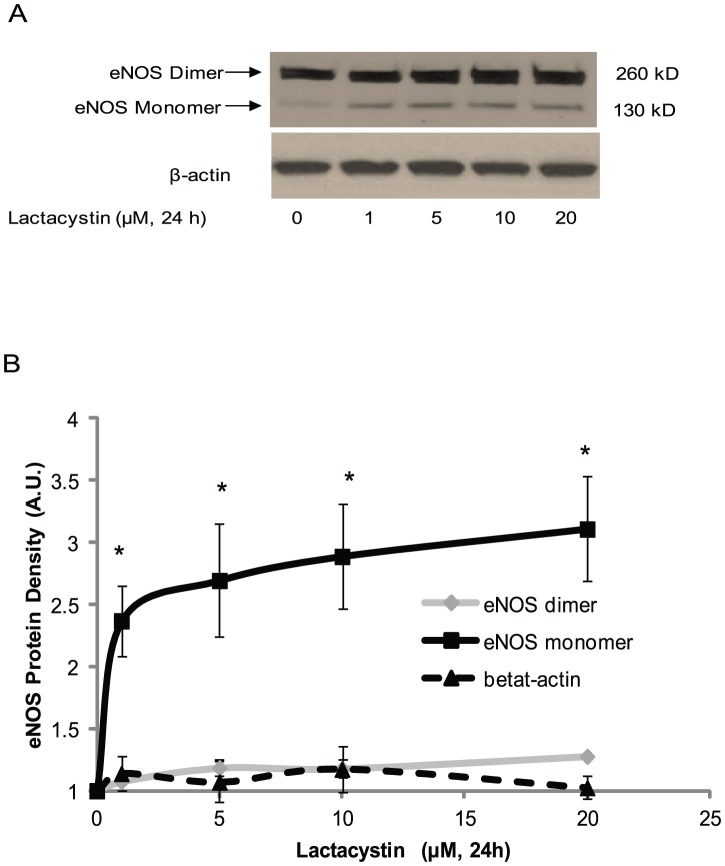
Inhibition of proteasome 26S resulted in an eNOS monomer accumulation in BAECs. BAECs were incubated with indicated concentration of lactacystin overnight. The protein was harvested with RIPA buffer and run on SDS-PAGE gel at low temperature (4°C) and the eNOS monomer and dimer content was assessed by western blot and quantified using densitometry. (A) Lactacystin caused a dose-dependent accumulation of eNOS monomer but not dimer; (B) The eNOS monomer and dimer in BAECs were normalized with the controls (0 h) and represented as mean ± SE from four independent experiments. * P<0.05 comparison with control.

To further investigate the functionality of eNOS monomer or dimer forms, we studied the eNOS monomer and dimer content in BAECs with low temperature gel electrophoresis. In BAECs at normal cultured condition, eNOS existed predominantly as a dimer (MW 260 kDa), with only trace eNOS monomers present (MW 130 kDa) (Figure 2A). Lactacystin, an inhibitor of the 26S proteasome, caused a dose-dependent accumulation of eNOS monomers that reached a maximum at a concentration of 20 µM. In contrast, lactacystin caused only a minimum increase in eNOS dimer in BAECs (Figure 2B). These results indicate that eNOS also exists as both dimer and monomer *in vivo* with eNOS monomers being eliminated by a high efficiency proteasome system.

**Figure 3:**
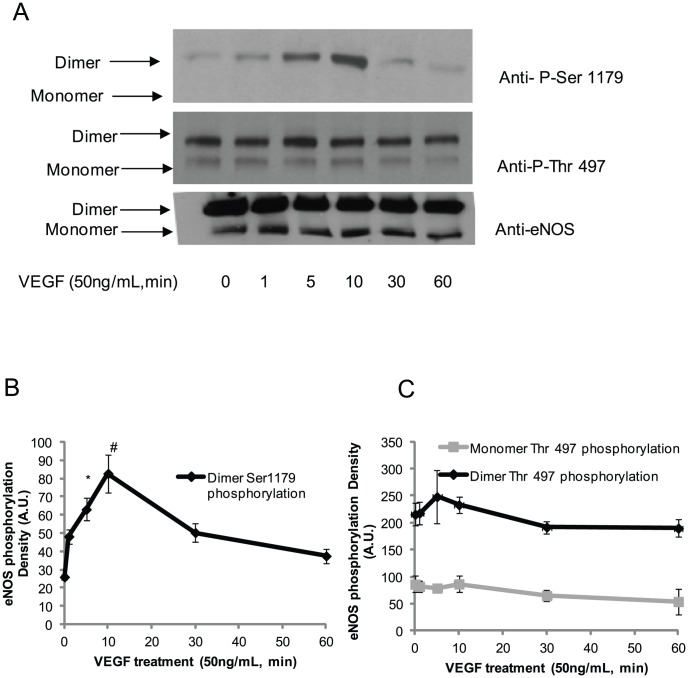
VEGF changed the Serine 1179 phosphorylation status in dimers only and did not alter the phosphorylation status of Threonine 497 in eNOS dimers or monomers. (A) After serum starvation for two hours, BAECs were treated with VEGF (50 ng/ml) for indicated time. The cell lysates were run on SDS-PAGE gels at low temperature. After transferring the protein to nitrocellulose membranes, they were probed with the indicated antibodies. (B) eNOS dimer Ser1179 phosphorylation density was determined by AlphaImager and normalized with control. The results were representative of three independent experiments. (C) The Thr497 phosphorylation density on eNOS dimer and monomer was determined by AlphaImager and normalized with control. The results were represented from three independent experiments (*P<0.05 comparing to control).

Previous reports have demonstrated that VEGF regulates eNOS activity, which has effects on multiple endothelial functions, including barrier integrity and angiogenesis [33], [34]. Our data indicate that Ser1179 and Thr497 phosphorylation alter eNOS activity in purified eNOS. As a typical tyrosine receptor kinase agonist, VEGF–mediated Ser1179 phosphorylation is accompanied by an increase eNOS activity [32]. Thus, by using VEGF-mediated eNOS phosphorylation as model, we next aimed to determine whether eNOS Ser1179 phosphorylation changed its dimer and monomer ratio in BAECs. The effects of VEGF on eNOS phosphorylation in BAECs were assessed in time course. We found that Ser1179 phosphorylation existed exclusively in the dimer form and VEGF induced a time-dependent eNOS Ser1179 phosphorylation that reached a maximum at 10 minutes, consistent with previous reports (Figure 3A top panel and Figure 3B) [35]. However, VEGF did not alter the Thr497 phosphorylation status in BAECs (Figure 3A middle panel and Figure 3C). Moreover, Ser1179 phosphorylation was only observed in eNOS dimer, whereas Thr497 phosphorylation was observed in both eNOS dimers and monomers. Similar to the dephosphorylation induced by PP2A and PP1, the changes in eNOS phosphorylation status induced by VEGF did not change eNOS dimer∶monomer ratio in BAECs (Figure 3A bottom panel).

**Figure 4:**
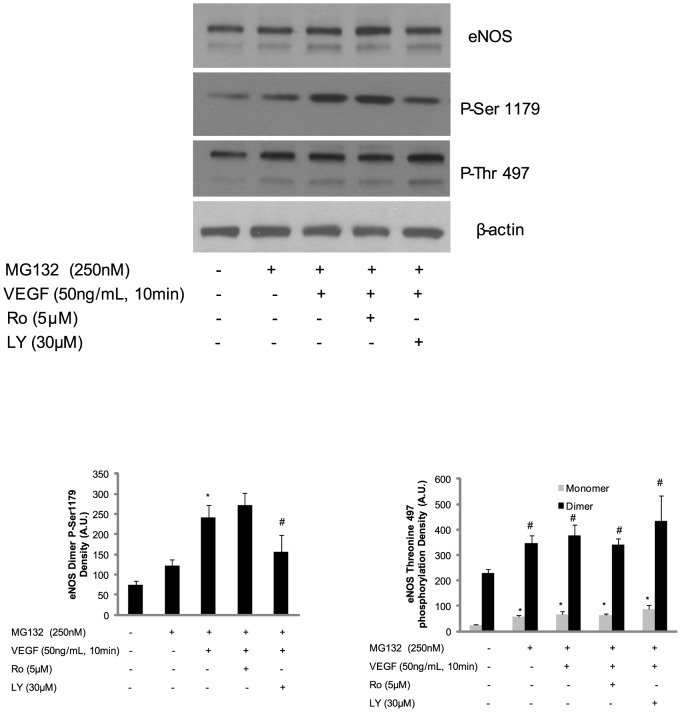
The PI3K signal pathway is the major pathway causing eNOS Serine 1179 phosphorylation induced by VEGF. After BAECs were treated with MG132 and Ro318220 or LY294002 overnight followed by VEGF challenge, the cell lysates were harvested for the western blotting. The eNOS Ser1179 and Thr497 phosphorylation density were determined by AlphaImager. The results are representative of three independent experiments. (*P<0.05 by one way ANOVA for monomers relative to untreated control and #P<0.05 by one way ANOVA for dimers relative to untreated control).

Previous studies demonstrate that VEGF activates eNOS through multiple signaling pathways, including the PI3K-Akt pathway and the PLC-PKC pathway [36], [37]. Thus, we next aimed to determine whether VEGF affects eNOS dimer or monomer and determine which signaling pathways are involved in regulation of Ser1179 and Thr497 phosphorylation. Treatment with the proteasome inhibitor MG132 caused an increase in eNOS monomers in BAECs. Additionally, this treatment increased eNOS Ser1179 in dimer and Thr497 phosphorylation in both eNOS dimer and monomer (Figure 4 second and third panel). Inhibition of PKC with Ro318220 did not alter VEGF-mediated eNOS Ser1179 and Thr497 phosphorylation. In contrast, inhibition of PI3K with LY294002 was found to decrease VEGF-mediated Ser1179 phosphorylation in dimer only (Figure 4A second panel). Interestingly, blockade of the VEGF-induced signaling pathway with LY294002 did not change the phosphorylation status of Thr497 in eNOS monomer or dimer (Figure 4A third panel). Notably, the blockade of these signaling pathways did not change eNOS dimer∶monomer ratio.

**Figure 5:**
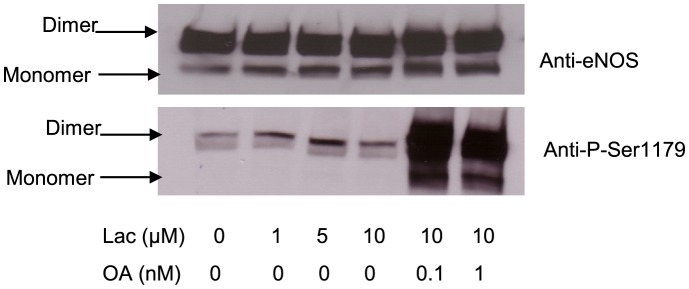
Inhibition of PP2A increased phosphorylation of Serine 1179 in eNOS dimer, but did not alter the eNOS dimer∶monomer ratio in BAECs. BAECs were treated with lactacystin or okadaic acid overnight and the samples were run on SDS-PAGE gel at low temperature (4°C) followed by western blot to determine eNOS phosphorylation and dimer∶monomer ratio as described in the Materials and Methods.

Additionally we utilized lactacystin to induce eNOS monomer accumulation in BAECs. This treatment caused an increase in Ser1179 phosphorylation in eNOS dimers, but not monomers (Figure 5). This Ser1179 phosphorylation increase is consistent with the dynamic balance between eNOS monomer and dimer. Lactacystin caused an accumulation of eNOS monomer and ultimately increased eNOS dimer as a feedback effect, although this increase was not significant (Figure 2). Similar to the effect of VEGF on eNOS phosphorylation in dimers, inhibition of PP2A with okadaic acid majorly affected eNOS dimer Ser1179 phosphorylation in BAECs (Figure 5).

**Figure 6:**
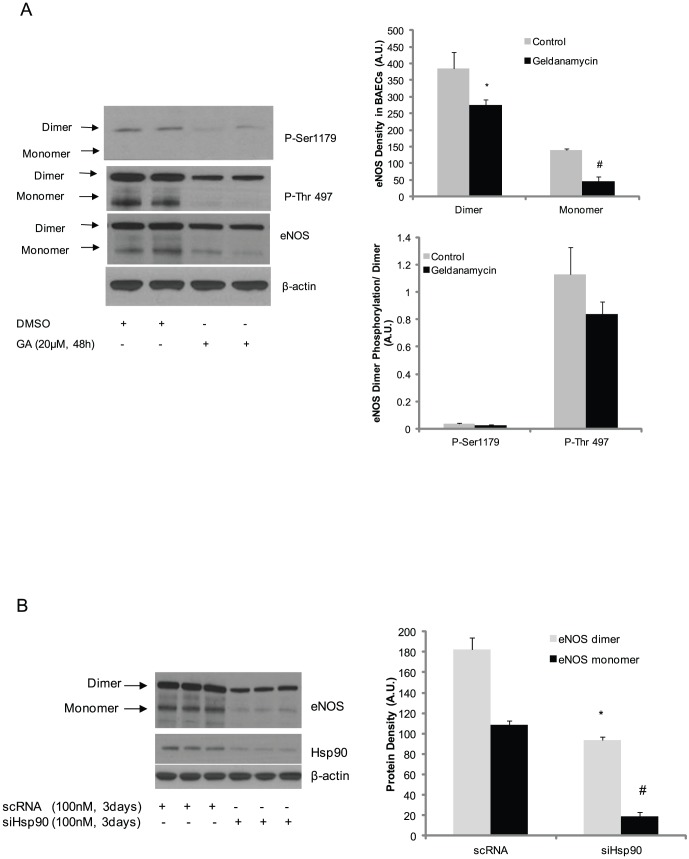
Inhibition of Hsp90 resulted in eNOS monomerization without change phosphorylation status of Serine 1179 and Threonine 497 in eNOS dimmer. (A) BAECs were treated with 20 µM geldanamycin for 48 hours and the samples were run SDS-PAGE gel at low temperature and blotted with the indicated antibodies to determine the changes in phosphorylation status of eNOS and dimers and monomers. The eNOS dimer and monomer densities were determined using AlphaImager. And The Ser1179 and Thr497 phosphorylation density on eNOS dimer were also determined using AlphaImager. All results are representative of three independent experiments. * and # P<0.05 comparison with control. (B) BAECs were silenced with scramble siRNA control (scRNA) or Hsp90 (siHsp90) as indicated concentration for 3 days, the cell lysates were blotted with eNOS or Hsp90 antibody respectively. The eNOS monomer and dimer density were measured using AlphaImager and the average of three independent experiments was presented (* and #P<0.05 compared to eNOS dimer and monomer control respectively).

It is established that heat shock protein 90 (Hsp90) plays an important role in the regulation of eNOS function [38]. However, it is unknown whether Hsp90 specifically targets either eNOS dimers or monomers and how it affects the phosphorylation status of eNOS. To address this, we inhibited Hsp90 with geldanamycin and measured the eNOS dimer∶monomer ratio and the phosphorylation status of Ser1179 and Thr497. We found that Hsp90 inhibition decreased both eNOS dimer and monomer (Figure 6A) and caused an accompanying dephosphorylation of Ser1179 and Thr497. The phosphorylation of Thr497 and Ser1179 in eNOS dimer was intact in the presence of geldanamycin compared to control (Figure 6A). Finally, to characterize the importance of Hsp90 for eNOS dimer structure, we silenced Hsp90 (siHsp90) and assessed eNOS dimer and monomer in BAECs. Similar to the effect of geldanamycin treatment, silencing Hsp90 decreased eNOS dimer content compared to scramble siRNA control (scRNA). Interestingly, silencing Hsp90 also caused a decrease in eNOS monomer content compared to scramble siRNA control (scRNA) (Figure 6B).

## Discussion

In the current study, we demonstrated that eNOS monomer, rather than dimer, is degraded by the 26S proteasome. We also confirmed that the phosphorylation status of Ser1179 and Thr497 alters eNOS activity. Additionally, we also found that the phosphorylation status of Ser1179 and Thr497 does not change eNOS dimer∶monomer ratio in cultured BAECs or in purified bovine eNOS. Finally, we confirmed the important role of Hsp90 in the regulation of eNOS structure and function. Previous studies have established that eNOS exists in dimer or monomer under various conditions [23]. However, although dimeric eNOS is thought to be the active form, this has not yet been conclusively proven due to the technical difficulties of isolating the dimers or monomers. Additionally, the mechanisms of eNOS monomerization and degradation have not yet been well characterized. Previous studies in BAECs have demonstrated that inhibition of the proteasome with MG132 causes an accumulation of eNOS, which leads to increased eNOS activity [39], [40]. However, it still has not determined which form of eNOS accumulates in cells and how this affects eNOS activity. Using a low-temperature gel technique, we found that proteasome inhibition causes a selective increase in eNOS monomers in BAECs. This increase in eNOS monomer may further affect eNOS monomer∶dimer ratio and activity [40].

Increasing evidence indicates that Ser1177 of eNOS (bovine Ser1179) plays an important role in regulation of eNOS activity, making it a target for various biologically relevant agonists [2], [30]. We found that phosphorylation of Ser1179 or its mutation to aspartic acid results in an increase in bovine eNOS NO generation. Additionally, Ser1179 phosphorylation results in electron transfer efficiency in eNOS reductase domain [41]. Here, we demonstrate that Ser1179 phosphorylation exclusively exists in eNOS dimer at both resting and stimulated condition (Figures 2 and 3). This phenomenon indicates that the dimer structure of eNOS is the predominant form which is the subject to Ser1179 phosphorylation. However, Ser1179 phosphorylation was detected on both eNOS monomer and dimer in purified eNOS (Figure 1A). This discrepancy of Ser1179 phosphorylation status between eNOS *in vitro* and purified eNOS indicates that BAECs have a sophisticated mechanism for Ser1179 dephosphorylation that is not present in E. coli. Moreover, Ser1179 phosphorylation status does not change the eNOS dimer∶monomer ratio in purified eNOS or in BAEC eNOS. Finally, VEGF-mediated eNOS phosphorylation also demonstrated that the phosphorylation status of Ser1179 does not change the eNOS dimer and monomer ratio in BAECs. However, inhibition of eNOS degradation not only increased both eNOS monomer and dimer but also increased phosphorylation of Ser1179 in dimer (Figures 4 and 5). These results indicate that eNOS dimerization is necessary for amino acid modification, such as Ser1179 phosphorylation.

Previous studies have demonstrated that Thr497 functions as a switch for eNOS binding with Ca^2+^, CaM complex [42]. Additionally, it has been reported that bradykinin treatment induces Thr497 dephosphorylation and enhances Ca^2+^/CaM binding with eNOS in BAECs [43]. Similar to Ser1179, the phosphorylation status of Thr497 does not change the dimer∶monomer ratio in purified eNOS or BAEC eNOS. However, contrary to previously published observations, our data indicate that dephosphorylation of Thr497 causes a decrease in eNOS activity and superoxide generation in purified eNOS [9]. This could be due to the fact that alterations of the phosphorylation status of Thr497 in cells may involve more complicated mechanisms, such as dephosphorylation of other critical amino acids as dephosphorylation of eNOS Thr497. Contrary to Ser1179 phosphorylation, Thr497 phosphorylation was found in both eNOS dimers and monomers under unstimulated conditions. Furthermore, VEGF did not induce Thr497 phosphorylation changes in both eNOS dimer and monomer indicating that either the sensitivity or the mechanism of phosphorylation differs from that of Ser1179 (Figure 3A middle panel and Figure 4) [9].

Lastly, previous studies demonstrate that Hsp90 plays an important role in maintaining eNOS activity and dimeric structure [19]. Consistent with previous observations, our data indicate that inhibition of Hsp90 with geldanamycin or silencing of Hsp90 destabilizes eNOS dimers and results in eNOS degradation. However, normalization of phosphorylation of Thr497 and Ser1179 with eNOS dimer demonstrate that the phosphorylation status of both Ser1179 and Thr497 do not change in eNOS dimer (Figure 6A) [44]. These results indicate that the eNOS phosphorylation status is intact in eNOS dimer in the presence of geldanamycin and that the eNOS dimer structure is the major factor that determines the maintenance of eNOS phosphorylation. Finally, Hsp90 plays important role in maintenance of eNOS dimer structure. Inhibition of Hsp90 with geldanamycin or silencing of Hsp90 causes the eNOS dimer to dissociate into monomers and exposes Ser1179 and Thr497 on the monomers to various phosphatases, resulting in eNOS dephosphorylation [44].

In conclusion, our findings indicate that eNOS monomers, but not dimers, are degraded by the 26S proteasome. Using purified eNOS and eNOS in BAECs, we demonstrate that dephosphorylation of Ser1179 or Thr497 decreases eNOS activity without affecting its structural stability. VEGF and phosphatase induced changes in the phosphorylation status of eNOS do not alter eNOS dimerization. However, the change in the eNOS dimer∶monomer ratio by proteasome inhibition was accompanied by a change in eNOS phosphorylation. Finally, inhibition of Hsp90 causes eNOS monomerization, accompanied by eNOS degradation. Thus, we have characterized, for the first time, the complicated relationship of eNOS structure, amino acid modification and function. These studies provide novel insights into the mechanisms underlying eNOS dimerization, posttranslational modifications and eNOS activity. Due to the involvement of eNOS in multiple disease states, these studies may help inform future translational research aimed at developing therapeutics to alter eNOS functioning.

## References

[1] Di Lorenzo A, Lin MI, Murata T, Landskroner-Eiger S, Schleicher M, et al.. (2013) eNOS derived nitric oxide regulates endothelial barrier function via VE cadherin and Rho GTPases. J Cell Sci. 10.1242/jcs.115972 PMC3860306 24046447

[2] Atochin DN, Wang A, Liu VW, Critchlow JD, Dantas AP, et al (2007) The phosphorylation state of eNOS modulates vascular reactivity and outcome of cerebral ischemia in vivo. J Clin Invest 117: 1961–1967. 17557122 10.1172/JCI29877 PMC1884686

[3] Hatakeyama T, Pappas PJ, Hobson RW 2nd, Boric MP, Sessa WC, et al (2006) Endothelial nitric oxide synthase regulates microvascular hyperpermeability in vivo. J Physiol 574: 275–281. 16675496 10.1113/jphysiol.2006.108175 PMC1817804

[4] Alderton WK, Cooper CE, Knowles RG (2001) Nitric oxide synthases: structure, function and inhibition. Biochem J 357: 593–615. 11463332 10.1042/0264-6021:3570593 PMC1221991

[5] Rafikov R, Fonseca FV, Kumar S, Pardo D, Darragh C, et al (2011) eNOS activation and NO function: structural motifs responsible for the posttranslational control of endothelial nitric oxide synthase activity. J Endocrinol 210: 271–284. 21642378 10.1530/JOE-11-0083 PMC3326601

[6] Andrew PJ, Mayer B (1999) Enzymatic function of nitric oxide synthases. Cardiovasc Res 43: 521–531. 10690324 10.1016/s0008-6363(99)00115-7

[7] Sorenson J, Santhanam AV, Smith LA, Akiyama M, Sessa WC, et al (2005) Expression and function of recombinant S1179D endothelial NO synthase in human pial arteries. Stroke 36: 158–160. 15569867 10.1161/01.STR.0000150489.47080.67

[8] Akiyama M, Eguchi D, Weiler D, O'Brien T, Kovesdi I, et al (2002) Expression and function of recombinant S1179D endothelial nitric oxide synthase in canine cerebral arteries. Stroke 33: 1071–1076. 11935063 10.1161/hs0402.105553

[9] Harris MB, Ju H, Venema VJ, Liang H, Zou R, et al (2001) Reciprocal phosphorylation and regulation of endothelial nitric-oxide synthase in response to bradykinin stimulation. J Biol Chem 276: 16587–16591. 11340086 10.1074/jbc.M100229200

[10] Xu H, Shi Y, Wang J, Jones D, Weilrauch D, et al (2007) A heat shock protein 90 binding domain in endothelial nitric-oxide synthase influences enzyme function. J Biol Chem 282: 37567–37574. 17971446 10.1074/jbc.M706464200

[11] Schulz E, Jansen T, Wenzel P, Daiber A, Munzel T (2008) Nitric oxide, tetrahydrobiopterin, oxidative stress, and endothelial dysfunction in hypertension. Antioxid Redox Signal 10: 1115–1126. 18321209 10.1089/ars.2007.1989

[12] Cai S, Khoo J, Channon KM (2005) Augmented BH4 by gene transfer restores nitric oxide synthase function in hyperglycemic human endothelial cells. Cardiovasc Res 65: 823–831. 15721862 10.1016/j.cardiores.2004.10.040

[13] Taverne YJ, de Beer VJ, Hoogteijling BA, Juni RP, Moens AL, et al (1985) Nitroso-redox balance in control of coronary vasomotor tone. J Appl Physiol (1985) 112: 1644–1652. 10.1152/japplphysiol.00479.2011 22362403

[14] Chen W, Druhan LJ, Chen CA, Hemann C, Chen YR, et al (2010) Peroxynitrite induces destruction of the tetrahydrobiopterin and heme in endothelial nitric oxide synthase: transition from reversible to irreversible enzyme inhibition. Biochemistry 49: 3129–3137. 20184376 10.1021/bi9016632 PMC2851177

[15] Antoniades C, Shirodaria C, Warrick N, Cai S, de Bono J, et al (2006) 5-methyltetrahydrofolate rapidly improves endothelial function and decreases superoxide production in human vessels: effects on vascular tetrahydrobiopterin availability and endothelial nitric oxide synthase coupling. Circulation 114: 1193–1201. 16940192 10.1161/CIRCULATIONAHA.106.612325

[16] Venema RC, Ju H, Zou R, Ryan JW, Venema VJ (1997) Subunit interactions of endothelial nitric-oxide synthase. Comparisons to the neuronal and inducible nitric-oxide synthase isoforms. J Biol Chem 272: 1276–1282. 8995432 10.1074/jbc.272.2.1276

[17] Persechini A, Tran QK, Black DJ, Gogol EP (2013) Calmodulin-induced structural changes in endothelial nitric oxide synthase. FEBS Lett 587: 297–301. 23266515 10.1016/j.febslet.2012.12.012 PMC3569036

[18] Byun EB, Ishikawa T, Suyama A, Kono M, Nakashima S, et al (2012) A procyanidin trimer, C1, promotes NO production in rat aortic endothelial cells via both hyperpolarization and PI3K/Akt pathways. Eur J Pharmacol 692: 52–60. 22796647 10.1016/j.ejphar.2012.07.011

[19] Ramirez V, Mejia-Vilet JM, Hernandez D, Gamba G, Bobadilla NA (2008) Radicicol, a heat shock protein 90 inhibitor, reduces glomerular filtration rate. Am J Physiol Renal Physiol 295: F1044–1051. 18667483 10.1152/ajprenal.90278.2008

[20] Komers R, Schutzer WE, Reed JF, Lindsley JN, Oyama TT, et al (2006) Altered endothelial nitric oxide synthase targeting and conformation and caveolin-1 expression in the diabetic kidney. Diabetes 55: 1651–1659. 16731827 10.2337/db05-1595

[21] Kolodziejski PJ, Musial A, Koo JS, Eissa NT (2002) Ubiquitination of inducible nitric oxide synthase is required for its degradation. Proc Natl Acad Sci U S A 99: 12315–12320. 12221289 10.1073/pnas.192345199 PMC129442

[22] Kolodziejski PJ, Rashid MB, Eissa NT (2003) Intracellular formation of "undisruptable" dimers of inducible nitric oxide synthase. Proc Natl Acad Sci U S A 100: 14263–14268. 14614131 10.1073/pnas.2435290100 PMC283580

[23] Benson MA, Batchelor H, Chuaiphichai S, Bailey J, Zhu H, et al (2013) A Pivotal Role for Tryptophan 447 in Enzymatic Coupling of Human Endothelial Nitric Oxide Synthase (eNOS): EFFECTS ON TETRAHYDROBIOPTERIN-DEPENDENT CATALYSIS AND eNOS DIMERIZATION. J Biol Chem 288: 29836–29845. 23965989 10.1074/jbc.M113.493023 PMC3795282

[24] Zhang QJ, Holland WL, Wilson L, Tanner JM, Kearns D, et al (2012) Ceramide mediates vascular dysfunction in diet-induced obesity by PP2A-mediated dephosphorylation of the eNOS-Akt complex. Diabetes 61: 1848–1859. 22586587 10.2337/db11-1399 PMC3379648

[25] Ruan L, Torres CM, Buffett RJ, Kennard S, Fulton D, et al (2013) Calcineurin-mediated dephosphorylation of eNOS at serine 116 affects eNOS enzymatic activity indirectly by facilitating c-Src binding and tyrosine 83 phosphorylation. Vascul Pharmacol 59: 27–35. 23727078 10.1016/j.vph.2013.05.004 PMC3824616

[26] Zhang QJ, Holland WL, Wilson L, Tanner JM, Kearns D, et al (2012) Ceramide mediates vascular dysfunction in diet-induced obesity by PP2A-mediated dephosphorylation of the eNOS-Akt complex. Diabetes 61: 1848–1859. 22586587 10.2337/db11-1399 PMC3379648

[27] Vasquez-Vivar J, Kalyanaraman B, Martasek P, Hogg N, Masters BS, et al (1998) Superoxide generation by endothelial nitric oxide synthase: the influence of cofactors. Proc Natl Acad Sci U S A 95: 9220–9225. 9689061 10.1073/pnas.95.16.9220 PMC21319

[28] Komarov AM, Wink DA, Feelisch M, Schmidt HH (2000) Electron-paramagnetic resonance spectroscopy using N-methyl-D-glucamine dithiocarbamate iron cannot discriminate between nitric oxide and nitroxyl: implications for the detection of reaction products for nitric oxide synthase. Free Radic Biol Med 28: 739–742. 10754269 10.1016/s0891-5849(00)00156-8

[29] Gopalakrishnan B, Nash KM, Velayutham M, Villamena FA (2012) Detection of nitric oxide and superoxide radical anion by electron paramagnetic resonance spectroscopy from cells using spin traps. J Vis Exp: e2810. 10.3791/2810 PMC3486746 22929836

[30] Garcia C, Nunez-Anita RE, Thebault S, Arredondo Zamarripa D, Jeziorsky MC, et al.. (2013) Requirement of phosphorylatable endothelial nitric oxide synthase at Ser-1177 for vasoinhibin-mediated inhibition of endothelial cell migration and proliferation in vitro. Endocrine. 10.1007/s12020-013-9964-4 23640371

[31] Scotland RS, Morales-Ruiz M, Chen Y, Yu J, Rudic RD, et al (2002) Functional reconstitution of endothelial nitric oxide synthase reveals the importance of serine 1179 in endothelium-dependent vasomotion. Circ Res 90: 904–910. 11988492 10.1161/01.res.0000016506.04193.96

[32] Lin MI, Fulton D, Babbitt R, Fleming I, Busse R, et al (2003) Phosphorylation of threonine 497 in endothelial nitric-oxide synthase coordinates the coupling of L-arginine metabolism to efficient nitric oxide production. J Biol Chem 278: 44719–44726. 12952971 10.1074/jbc.M302836200

[33] Di Lorenzo A, Lin MI, Murata T, Landskroner-Eiger S, Schleicher M, et al.. (2013) eNOS derived nitric oxide regulates endothelial barrier function via VE cadherin and Rho GTPases. J Cell Sci. 10.1242/jcs.115972 PMC3860306 24046447

[34] Bhat TA, Nambiar D, Tailor D, Pal A, Agarwal R, et al (2013) Acacetin Inhibits In Vitro and In Vivo Angiogenesis and Downregulates Stat Signaling and VEGF Expression. Cancer Prev Res (Phila) 6: 1128–1139. 23943785 10.1158/1940-6207.CAPR-13-0209 PMC3808880

[35] Chen Y, Medhora M, Falck JR, Pritchard KA Jr, Jacobs ER (2006) Mechanisms of activation of eNOS by 20-HETE and VEGF in bovine pulmonary artery endothelial cells. Am J Physiol Lung Cell Mol Physiol 291: L378–385. 16679377 10.1152/ajplung.00424.2005

[36] Feliers D, Chen X, Akis N, Choudhury GG, Madaio M, et al (2005) VEGF regulation of endothelial nitric oxide synthase in glomerular endothelial cells. Kidney Int 68: 1648–1659. 16164642 10.1111/j.1523-1755.2005.00575.x

[37] Gelinas DS, Bernatchez PN, Rollin S, Bazan NG, Sirois MG (2002) Immediate and delayed VEGF-mediated NO synthesis in endothelial cells: role of PI3K, PKC and PLC pathways. Br J Pharmacol 137: 1021–1030. 12429574 10.1038/sj.bjp.0704956 PMC1573579

[38] Cortes-Gonzalez C, Barrera-Chimal J, Ibarra-Sanchez M, Gilbert M, Gamba G, et al (2010) Opposite effect of Hsp90alpha and Hsp90beta on eNOS ability to produce nitric oxide or superoxide anion in human embryonic kidney cells. Cell Physiol Biochem 26: 657–668. 21063103 10.1159/000322333

[39] Govers R, de Bree P, Rabelink TJ (2003) Involvement of the proteasome in activation of endothelial nitric oxide synthase. Life Sci 73: 2225–2236. 12927592 10.1016/s0024-3205(03)00644-1

[40] Stangl V, Lorenz M, Meiners S, Ludwig A, Bartsch C, et al (2004) Long-term up-regulation of eNOS and improvement of endothelial function by inhibition of the ubiquitin-proteasome pathway. Faseb J 18: 272–279. 14769821 10.1096/fj.03-0054com

[41] McCabe TJ, Fulton D, Roman LJ, Sessa WC (2000) Enhanced electron flux and reduced calmodulin dissociation may explain "calcium-independent" eNOS activation by phosphorylation. J Biol Chem 275: 6123–6128. 10692402 10.1074/jbc.275.9.6123

[42] Fleming I, Fisslthaler B, Dimmeler S, Kemp BE, Busse R (2001) Phosphorylation of Thr(495) regulates Ca(2+)/calmodulin-dependent endothelial nitric oxide synthase activity. Circ Res 88: E68–75. 11397791 10.1161/hh1101.092677

[43] Michell BJ, Harris MB, Chen ZP, Ju H, Venema VJ, et al (2002) Identification of regulatory sites of phosphorylation of the bovine endothelial nitric-oxide synthase at serine 617 and serine 635. J Biol Chem 277: 42344–42351. 12171920 10.1074/jbc.M205144200

[44] Kupatt C, Dessy C, Hinkel R, Raake P, Daneau G, et al (2004) Heat shock protein 90 transfection reduces ischemia-reperfusion-induced myocardial dysfunction via reciprocal endothelial NO synthase serine 1177 phosphorylation and threonine 495 dephosphorylation. Arterioscler Thromb Vasc Biol 24: 1435–1441. 15178564 10.1161/01.ATV.0000134300.87476.d1

